# Real-Time Imaging of Retinal Cell Apoptosis by Confocal Scanning Laser Ophthalmoscopy and Its Role in Glaucoma

**DOI:** 10.3389/fneur.2018.00338

**Published:** 2018-05-15

**Authors:** Elizabeth Yang, Toby S. Al-Mugheiry, Eduardo M. Normando, Maria F. Cordeiro

**Affiliations:** ^1^The Western Eye Hospital, Imperial College Healthcare NHS Trust, London, United Kingdom; ^2^The Imperial College Ophthalmic Research Group (ICORG), Imperial College London, London, United Kingdom; ^3^Queen Elizabeth Hospital, King’s Lynn NHS Foundation Trust, Norfolk, United Kingdom; ^4^Insitute of Ophthalmology, University College London, London, United Kingdom

**Keywords:** glaucoma, annexin, retinal ganglion cells, imaging, apoptosis

## Abstract

Glaucoma is one of the leading causes of irreversible blindness in the world. It is characterized by the progressive loss of retinal ganglion cells (RGCs), mainly through the process of apoptosis. Glaucoma patients often come to clinical attention when irreversible loss of visual function has been already established; therefore, early recognition of RGC apoptosis is inordinately important in disease prevention. The novel technology called Detection of Apoptosing Retinal Cells (DARC) allows real-time *in vivo* quantification of apoptosing cells through the use of a fluorescent biomarker and a confocal scanning ophthalmoscope. A recent Phase I clinical trial has evaluated the safety of DARC and its ability to detect retinal apoptosis in glaucoma patients and healthy volunteers. Results suggest that DARC may have potential in the early detection of glaucoma, which could help alleviate the medical, social, and economic burden associated with this blinding condition.

## Introduction

Glaucoma is one of the leading causes of blindness with over 70 million people affected worldwide and projected to be 80 million by 2020 (1). It is as an optic neuropathy characterized by cupping of the optic disk, thinning of the retinal nerve fiber layer, and visual field loss (2), caused by the death of retinal ganglion cells (RGCs) (3) predominantly mediated by the mechanism of programmed cell death, otherwise known as apoptosis (4). During apoptosis, cascades of enzymes are activated leading to a series of biochemical and morphological changes, including cell and nuclear shrinkage, chromatin condensation, DNA fragmentation, and bleb formation (5). It is at the level of the cell membrane that one of the key mechanisms of apoptosis takes place: the translocation of phosphatidylserine (PS) from the inner to the outer leaflet of the cell membrane (6). Although exposed PS acts as an “eat me” signal to phagocytes, it is also where the endogenous molecule Annexin 5 (ANX) binds (7). This property has been exploited for many years in the detection of apoptosis using fluorescently labeled ANX and recently utilized in the development of a novel *in vivo* retinal imaging technology called Detection of Apoptosing Retinal Cells (DARC) technology (8–11). DARC has potential in the early identification of patients with glaucoma before irreversible damage occurs (8). This review summarizes the development of the DARC technology and discusses its role in glaucoma and neurodegeneration.

## Biomarkers and Apoptosis

The annexins are a family of Ca^2+^-dependent proteins which bind to anionic phospholipids (12). Annexin V is a protein with a high affinity for PS (13). Translocation of PS from the inner to the outer leaflet of the cellular membrane is an early event in apoptosis and its identification is a potential biomarker for early detection of cell death. ANX has been used for many years in diagnostic *in vitro* assays for the identification of apoptotic cells in laboratory samples. In the last two decades, ANX has been conjugated to radioactive materials, such as Technetium-99, and used clinically to detect apoptosis in many conditions including breast cancer (14), non-Hodgkin’s lymphoma (15), myocardial infarction (16), and rectal cancer (17).

## The Development of DARC

The technique known as DARC has been developed over the last few years to initially to assess the degree of RGC apoptosis in experimental models of glaucoma (11, 18). More recently, it has also been used to investigate the occurrence of retinal cell apoptosis in other neurodegenerative disease models (19–21).

Although the loss of RGCs underlies the pathogenesis of glaucoma and other optic neuropathies, its degeneration is known to occur many years before any visual field defects are identified (22, 23). Patients are typically managed by serial measurements of intraocular pressure (IOP), retinal nerve fiber layer and optic disk imaging, as well as with visual field tests. However, these parameters alone may not be sufficient for monitoring glaucoma progression, as progressive visual field loss has been demonstrated in patients despite “well-controlled” IOP (24, 25). The gold standard for detecting glaucomatous changes remains standard automated white-on-white perimetry. It is a test that requires good patient cooperation, is time consuming, and can be inconsistent and variably interpreted (26). Visual field changes may not become apparent until the disease is well established and by then, irreversible damage has occurred.

**Figure 1:**
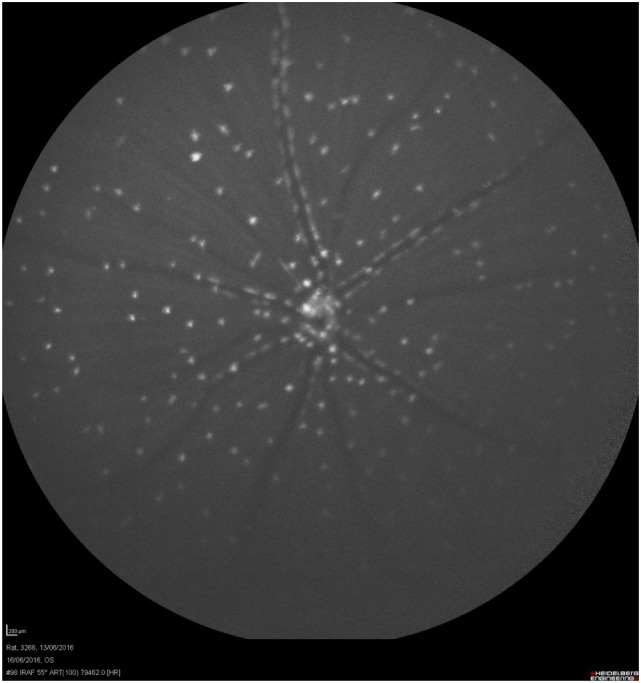
Visualization of nerve cell apoptosis. Retinal ganglion cell (RGC) apoptosis could be detected *in vivo* by using a modified cLSO with an argon laser (488 nm) for illumination and a wide band-pass filter with short-wavelength cutoff of a 521-nm filter and intravitreal Alexa-Fluor 488 fluorescent-labeled annexin 5. This was a model of staurosporine (SSP)-induced apoptosis. This rat’s eye was treated with intravitreal SSP (0.5 µg). This retinal image shows extensive RGC apoptosis (white spots, annexin 5-positive apoptotic cells).

The first *in vivo* use of DARC technology was demonstrated in experimental glaucoma in 2004 (20) where real-time visualization of a single nerve cell apoptosis through the eye’s clear optical media was recorded (Figure 1). Validation of the technique was established using ocular hypertensive and optic nerve transection models (20). Histological analyses were used to confirm the *in vivo* findings. Repeated assessment of the same eye over time with DARC has also shown promising results for its application in evaluating potential neuroprotective strategies (27, 28).

Detection of apoptosing retinal cell has been validated in a few other experimental models of neurodegenerative disease and optic neuropathy, where is has been used to assess disease severity and treatment efficacy. Some studies investigate different neuroprotective strategies in a rat model of glaucoma (28) as well as novel potential methods of reversing optic neuropathy (29) as summarized in the table below (Table 1).

**Table 1:** Summary of previous studies showing the use of DARC.

Optic neuropathy model	Title of study	Label	Findings	Reference
Rat	Real-time imaging of single nerve cell apoptosis in retinal neurodegeneration.	Anx with Alexa-Fluor 488 tag	Neuronal apoptosis in the eye of a living animal could be observed in real time using fluorescent-labeled annexin	Cordeiro et al. (20)
Glaucoma (OHT)				
Optic nerve transection	Proceedings of the National Academy of Science			

Rat glaucoma	Retinal ganglion cell (RGC) apoptosis in glaucoma is related to intraocular pressure (IOP) and IOP-induced effects on extracellular matrix	Anx with Alexa-Fluor 488 tag	RGC apoptosis in glaucoma strongly correlates with raised IOP and is significantly associated with IOP-induced changes in the RGC layer	Guo et al. (27)

Rat glaucoma	Assessment of neuroprotective effects of glutamate modulation on glaucoma-related RGC apoptosis *in vivo*	Anx with Alexa-Fluor 488 tag	RGC apoptosis was induced in rats by staurosporine (SSP) treatment. This novel SSP model was validated as a useful tool for screening neuroprotective strategies *in vivo*	Guo et al. (28)

Rat and mouse glaucoma (SSP-induced RGC cell death)	Assessment of rat and mouse RGC apoptosis imaging *in vivo* with different scanning laser ophthalmoscopes	Anx with Alexa-Fluor 488 tag	Fluorescent points (FPs) used as a measure of RGC apoptosis *in vivo* were detected in the mouse eye but only with the HRAII and not the Zeiss confocal scanning laser ophthalmoscope	Maass et al. (30)

Rat and mouse glaucoma	Imaging multiple phases of neurodegeneration: a novel approach to assessing cell death *in vivo*	Anx with Alexa-Fluor 488 tag or ANX776	An increase in retinal apoptosis induced by oxidative stress could be detected as a measure of disease activity	Cordeiro et al. (19)

Rat (DMSO solvent-induced retinal apoptosis)	Unexpected low-dose toxicity of the universal solvent, DMSO	ANX776	The study was to demonstrate that DMSO induced retinal apoptosis at low concentrations, with toxicity detected at levels >1%v/v using AnxA5	Galvao et al. (31)

Rat optic nerve transection model	Direct optic nerve sheath (DONS) application of Schwann cells prolongs RGC survival *in vivo*	Anx with Alexa-Fluor 488 tag or ANX776	A new method of delivery of Schwann cells for reversing optic neuropathy and used DARC to visualize RGC apoptosis as an outcome of successful rescue	Guo et al. (29)

Rat ischemia-reperfusion and partial optic nerve transection model	Adenosine A3 receptor activation is neuroprotective against retinal neurodegeneration	Anx with AlexaFluor 488 tag	DARC revealed a reduction of RGC apoptosis in the eyes treated with A3 agonist, compared to untreated control in partial optic nerve transection model—assessing its use as neuroprotective endpoint	Galvao et al. (32)

*Adapted from Cordeiro et al. (8). OHT, ocular hypertension*.

Over the years, the technology has evolved. To date, the DARC technique is composed of intravenous injection of an infrared fluorescently labeled ANX (ANX776), followed by retinal imaging using specific wavelengths (excitation: 786 nm; photodetector barrier filter: 800 nm; emission: 815 nm) using a commercially available confocal scanning laser ophthalmoscope (cSLO) and indocyanine green angiography settings. One of the key elements of the confocal imaging instrumentation is the ability to focus on the inner retina layers, which permits capturing of the signal coming from the RNFL and GCL layers (33–35).

Aside from DARC, other methods of imaging and monitoring cellular apoptosis have been studied. For example, TCapQ is an experimental technique which utilizes an intracellular near-infrared fluorescent peptide probe for the *in vivo* visualization of apoptotic RGCs. It involves using a cell-penetrating Tat-peptide conjugated to an effector caspase recognition sequence, a quencher and a fluorophore (Alexa-Fluor 488) that is activated by caspase 3 and 7 in the cytoplasm of apoptotic cells (36). This technology detects effector caspase activity, which signifies an apoptotic cascade activation (37). However, this method is complex and requires intracellular delivery of molecular imaging probes to enable detection of apoptosis. It also involves intravitreal administration, which is invasive and not the most suitable method for clinical translation.

AAV vectors are a method of targeting gene expression, which can be used for fluorescently labeling RGCs and studying RGC number *in vivo*. These vectors have the ability to incorporate different promoters, which allows for high specificity in targeting RGCs. AAV vectors are administered *via* intravitreal injections, with the aim of quantifying cell density *in vivo* (38). Again, intravitreal injections are invasive not the best option for monitoring patients. Also, the use of viral vectors and gene delivery is still associated with complications, which would make this an unsuitable test to use routinely in patients.

Adaptive optics scanning light ophthalmoscopy (AOSLO) is a method of imaging the RGC layer in the human retina without using any cell labeling techniques. It is possible to use this imaging method to study the RGC layer in glaucoma using its ability to visualize individual cells, although RGC imaging is difficult due to their low contrast edges. Subcellular resolution can also demonstrate the changes that subcellular structures undergo during apoptosis in glaucoma (39). However, AO imaging requires heavy image processing, and optimal images are very sensitive to focus position and difficult to obtain. Moreover, it is impossible to image the whole retina using AOSLO, as the field of view is very limited. This again makes it a suboptimal method for monitoring overall glaucoma progression.

Detection of apoptosing retinal cell allows for a less invasive, patient-acceptable method of imaging RGCs in a wide field of view in the retina, which could be clinically useful in monitoring progression in patients with optic neuropathy.

## Clinical Use of DARC

Early detection of any disease is paramount and is one of the most important aspects of any diagnostic test (40). In glaucoma, where functional visual field damage is only detectable after at least 40% of RGCs are lost, there is an unmet need for an early diagnostic tool (41). Apoptosis is a key process in glaucoma; therefore, its evaluation should be of utmost importance. Prior to DARC technology, the only method of assessing apoptosis definitively was through post mortem histology (22, 33). Experimental data from animal glaucoma models have shown the potential for DARC to predict the rate of disease progression in humans (20, 33, 34). DARC could provide a rapid and effective means of detecting apoptotic RGCs that would allow earlier detection and thus, acting as a biomarker for novel treatment strategies.

Clinical trials of DARC have involved the intravenous (IV) administration of ANX776 and an assessment of retinal cell apoptosis with commercially available cSLO devices. IV administration of angiographic contrast solutions, such as sodium fluorescein and indocyanine green, is a well-established method in ophthalmology for imaging retinal diseases (42). Use of IV radiolabeled Annexin V has been shown to be safe in multiple clinical trials and indications, including heart transplantation (43), breast cancer (14), non-Hodgkin’s lymphoma (14), and myocardial infarction (15).

**Figure 2:**
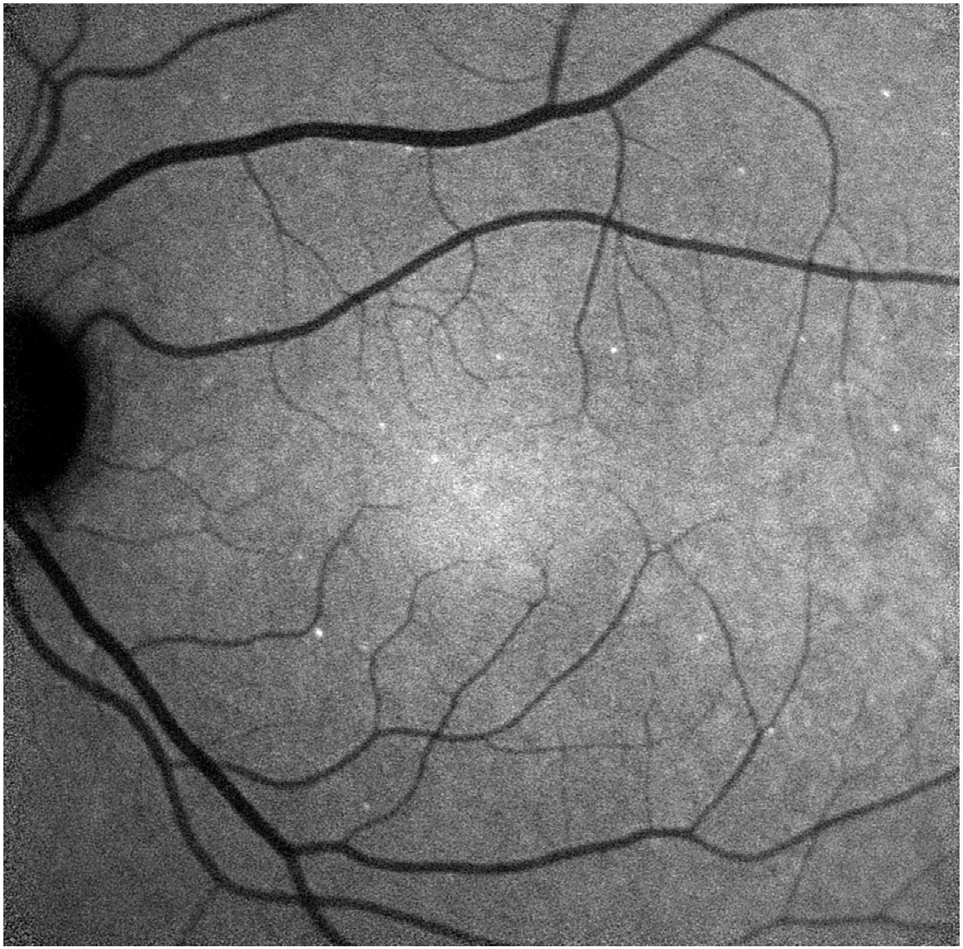
Detection of apoptosing retinal cell (DARC) count in a glaucoma patient. DARC counts are defined as new, unique individual ANX776-labeled spots, at their first appearance in the retina. ANX776 injections revealed single neuronal cell apoptosis in the retina of study subjects, and DARC counts are increased in affected glaucoma patients compared to healthy controls (8).

A recent Phase I study assessed the use of IV ANX776 to identify retinal cell apoptosis in humans (8). Briefly, the study has shown that apoptosing retinal cells can be seen in human patients as white fluorescent spots against a dark retinal background. The total number of spots makes up the DARC Count which gives a precise quantitative evaluation of cell damage at a given time point (Figure 2). In this Phase I trial, half the enrolled subjects were eight healthy volunteers and the other half were eight patients with progressive glaucoma. Although the trial was designed primarily to assess the safety and tolerability of ANX776 in patients, it showed that the DARC Count was significantly increased in glaucoma patients compared to healthy volunteers. Furthermore, the DARC Count correlated with increasing rates of glaucomatous progression. Importantly, ANX776 was shown to be safe and well tolerated with no serious adverse effects, such as anaphylaxis, arrhythmias, loss of consciousness, etc. There were single cases of mild and self-limiting adverse effects, for example, hematoma at the cannulation site and discomfort during phlebotomy, which were likely unrelated to ANX776. ANX776 is rapidly cleared from the human body and has a short half-life of about 10–36 min. The optimal dosing was determined based on the dose the better correlated with the highest level of DARC Counts (0.4 mg). Overall, the trial showed the potential of DARC as a method for detecting and assessing the level of neurodegeneration in glaucoma *in vivo* using RGC apoptosis as an indicator.

Currently, the DARC Phase II trial is being analyzed, having completed the patient intervention stage. It primarily aims to evaluate the efficacy of DARC in visualizing apoptotic retinal cells in patients with neurodegenerative diseases, including glaucoma, age-related macular degeneration, optic neuritis, and Down’s syndrome. Increasing evidence suggests the eye is involved in CNS diseases, such as Alzheimer’s disease (AD) and Parkinson’s diseases (PD). In particular it has been demonstrated, experimentally, that DARC can detect retinal cell death *in vivo* in a mouse model of AD (3XTG-AD) (19). Furthermore, DARC has been used to assess RGC apoptosis in an experimental model of PD, showing that retinal changes can be identified before classical histological changes in the brain occur (21).

## Future Directions

Detection of apoptosing retinal cell is currently being evaluated in clinical trials, and its validation will become increasingly important as it compared to existing gold standards in identifying and monitoring disease progression. Although not yet assessed clinically, experimentally DARC has been shown useful in testing treatment efficacy in a variety of different neurodegenerative conditions such as AD (44) and PD (19, 21, 28). In PD, it was shown that retinal changes may be a good surrogate biomarker of disease progression and treatment efficacy (21). Similarly, DARC technology could potentially be applied to studying rare but visually devastating mitochondrial optic neuropathies such as Leber’s hereditary optic neuropathy and dominant optic neuropathy (45). In the next few years, it is anticipated that DARC will be investigated in clinical treatment trials with an exploratory endpoint.

As DARC continues to develop, its application to glaucoma as well as other optic neuropathies and neurodegenerative disease represents a novel *in vivo* approach to assessing disease progression. Earlier detection of glaucoma could potentially allow for more efficacious treatment, and reduce the global health burden of this leading cause of blindness.

## Author Contributions

EY and TA-M drafted the article. EY, EN, and MC edited the article for submission.

## Conflict of Interest Statement

MC has a patent application concerning DARC technology. The other authors declare that the research was conducted in the absence of any commercial or financial relationships that could be construed as a potential conflict of interest.
